# MORF9 Functions in Plastid RNA Editing with Tissue Specificity

**DOI:** 10.3390/ijms20184635

**Published:** 2019-09-19

**Authors:** Faan Tian, Jinfa Yu, Ya Zhang, Yakun Xie, Binghua Wu, Ying Miao

**Affiliations:** Fujian Provincial Key Laboratory of Plant Functional Biology, College of Life Sciences, Fujian Agriculture and Forestry University, 350002 Fuzhou, China; 1160516017@fafu.edu.cn (F.T.); 1180514071@fafu.edu.cn (J.Y.); 1160539009@fafu.edu.cn (Y.Z.); yakun.xie@fafu.edu.cn (Y.X.); Binghua.wu@fafu.edu.cn (B.W.)

**Keywords:** organelles, RNA editing, tissue-specific, MORF9, *Arabidopsisthaliana*

## Abstract

RNA editing in plant mitochondria and plastids converts specific nucleotides from cytidine (C) to uridine (U). These editing events differ among plant species and are relevant to developmental stages or are impacted by environmental conditions. Proteins of the MORF family are essential components of plant editosomes. One of the members, MORF9, is considered the core protein of the editing complex and is involved in the editing of most sites in chloroplasts. In this study, the phenotypes of a T-DNA insertion line with loss of *MORF9* and of the genetic complementation line of *Arabidopsis* were analyzed, and the editing efficiencies of plastid RNAs in roots, rosette leaves, and flowers from the *morf9* mutant and the wild-type (WT) control were compared by bulk-cDNA sequencing. The results showed that most of the known *MORF9*-associated plastid RNA editing events in rosette leaves and flowers were similarly reduced by *morf9* mutation, with the exception that the editing rate of the sites *ndhB-872* and *psbF-65* declined in the leaves and that of *ndhB-586* decreased only in the flowers. In the roots, however, the loss of MORF9 had a much lower effect on overall plastid RNA editing, with nine sites showing no significant editing efficiency change, including *accD-794*, *ndhD-383*, *psbZ-50,* *ndhF-290*, *ndhD-878,* *matK-706*, *clpP1-559*, *rpoA-200*, and *ndhD-674*, which were reduced in the other tissues. Furthermore, we found that during plant aging, *MORF9* mRNA level, but not the protein level, was downregulated in senescent leaves. On the basis of these observations, we suggest that MORF9-mediated RNA editing is tissue-dependent and the resultant organelle proteomes are pertinent to the specific tissue functions.

## 1. Introduction

Post-transcriptional C to U RNA editing is widely present in transcripts of plant chloroplasts and mitochondria. It has been reported that in *Arabidopsis thaliana*,34 and 500 specific C loci were edited as U in plastids and in mitochondria, respectively [1,2]. Most edited RNA results in differently coded amino acids from those predicted by the genome. It is believed that RNA editing corrects mutations (T to C) in plant organelle genome [1,3,4].

The pentatricopeptide repeat (PPR) protein CRR4 was the first reported RNA editing factor in plants, in 2005 [5]. CRR4 had binding specificity to the transcript of *ndhD*, one of the chloroplast NAD(P)H dehydrogenase (NDH) complex-encoding gene, and converted the start codon ACG to AUG [5,6]. Later progress revealed that another group of proteins designed as MORF/RIP (multiple organellar RNA editing factor/RNA editing interaction protein) protein family also played important roles in RNA editing in plant organelles. The MORF family comprises nine members in *Arabidopsis*, namely, MORF1 to MORF9 [7]. Among them, MORF2 and MORF9 are localized in chloroplasts, whilst MORF1, MORF3, MORF4, MORF6, and MORF7 are mitochondrial proteins. The last two members, MORF5 and MORF8, are found in both chloroplasts and mitochondria [7]. Many proteins from the big PPR family were found to interact specifically with MORF proteins and to be required for plastid and/or mitochondria RNA editing [8,9,10,11,12,13,14,15]. Thus, of the term “editosome” was coined to indicate a protein complex that works as an RNA editing machinery [16]. For example, the interaction between MORF9 and PLS-type PPR proteins, forming the editosome complex, could improve significantly the binding efficiency between PPR proteins and the RNA substrate [17]. In recent years, a number of other novel proteins functioning in RNA editing were characterized, including PPO1 (protoporphrinogen IX oxidase 1), ORRMs (organelle RNA recognition motif-containing), and OZs (organelle zinc finger) [18,19,20,21,22,23,24]. As yet, MORF proteins are considered as the core components of organelle RNA editing complexes [7,25,26].

MORF9 is the most studied of the family and plays a role in multiple organelles, where its interaction with various PPR proteins was demonstrated [7,25,26]. RNA editing mediated by these multiple interactions affects plant growth and development and is involved in responses to environmental cues. In plastid, MORF9 was able to interact with MORF2 and MORF8 and edited at least 34 transcript sites within 24 genes [27]. The affected loci encode proteins with diverse functional implications in plant growth, development, and stress response, thus the editing was confirmed to be physiologically relevant [28,29,30,31,32]. However, the regulatory pattern of plastid multi-gene and multi-site editing associated with MORF9 in different tissues or organs is far from clear. In this study, we analyzed the phenotypes of a loss-of-MORF9 mutant, its complementation line, and an overexpression line and profiled the multiple RNA editing sites in different tissues and organs. We show that plastid RNA editing was differentially affected by MORF9 proteins in various tissues and organs, and the efficiency correlated specifically with the development of plant tissues and organs.

## 2. Results

### 2.1. Loss-of-MORF9 Protein Retarded Plant Growth and Reproductive Organ Development

**Figure 1:**
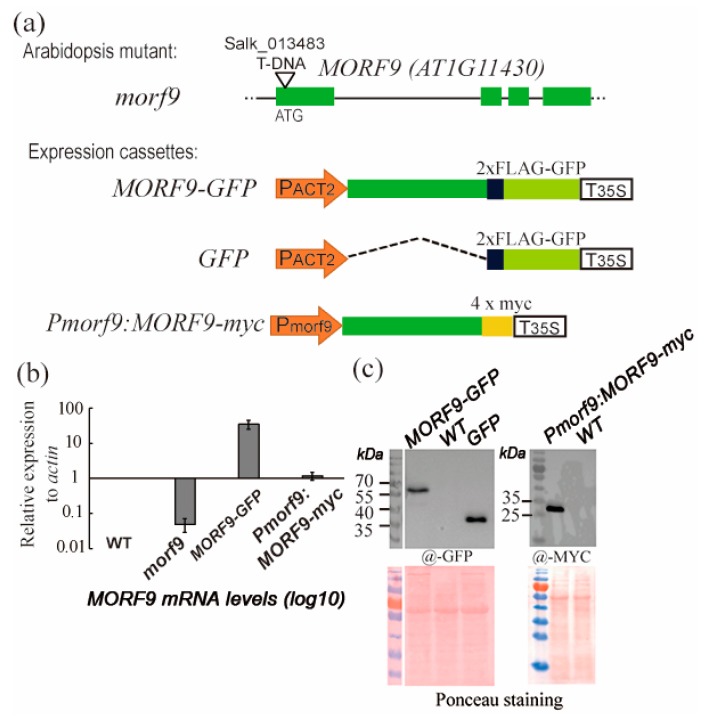
A T-DNA insertion line of *Arabidopsis* at the *MORF9* locus and generation of transgenic plants. (**a**) Schematic of the T-DNA insertion *morf9* mutant and the transgenic constructs for overexpression of a GFP-tagged MORF9 or GFP alone in wild-type (WT) plants and complementation (*Pmorf9:MORF9-myc*) of the T-DNA insertion *morf9* mutant. Not drawn to scale; (**b**) *MORF9* transcript levels in the *morf9* mutant and *MORF9* complementation plants by qRT-PCR. The relative transcript levels of *MORF9* with respect to *ACTIN* were normalized to those in WT plants. Error bars indicate the standard error (SE) of three biological replicates; (**c**) Western blots showing protein expression in the indicated plants using antibodies against GFP or MYC. The same membranes were stained with Ponceau for a loading control.

The homozygous T-DNA insertion line *morf9* (Figure 1a) was previously characterized as having defective leaf greening under light and developed eventually variegated leaf patterning at mature stage [7]. A complementation line with functional *MORF9* under its native promoter, *Pmorf9:MORF9-myc* (*ProMORF9:MORF9-4Xmyc*/*morf9*) (Figure 1a), was created by Huang [30]. Using these lines together with a transgenic line *MORF9-GFP* (*Pactin2:MORF9-2xflag-gfp*) on a WT background, we determined and compared *MORF9* gene expression at both transcript and protein levels. We observed that *MORF9* transcripts were hardly detected in the T-DNA insertion mutant *morf9*, whereas the overexpression transgenic plants produced 35-fold more abundant transcripts comparing to the WT plants in 21-day-old rosettes (Figure 1b). Further, recombinant MORF9 proteins were correctly detected in the overexpression and complementation lines (Figure 1c).

**Figure 2:**
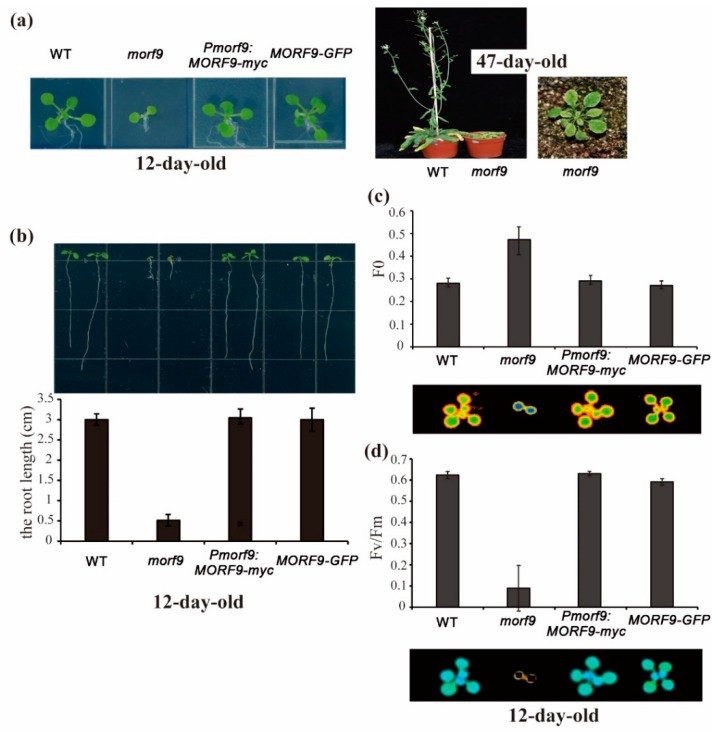
Phenotypes of *morf9* mutant and transgenic plants. (**a**) Plants at 12 days after germination in MS medium and at 35 days after transfer to soil; (**b**) Root morphogenesis of the *morf9* mutant and transgenic plants. The lower panel shows the average root length of 10 to 20 plants with standard errors; (**c**,**d**) Imaging and quantification of the leaf initiation fluorescence yield (F0) and efficiency of photosystem II (Fv/Fm) in plants with indicated genetic background at day 12 after germination, respectively. Data represent the mean of at least five plants with SE.

Phenotypically, *morf9* seedlings grown in sugar-free 1/2 MS medium showed a stunted plant, short roots, and yellow leaves (Figure 2a,b). These plants exhibited retarded growth with yellow variegated leaves after they were transferred to soil for 35 days (Figure 2a), the same phenotype as described by Takenaka [7]. At 12 days after germination (dag), the average root length of the *morf9* seedlings was one-sixth shorter than that of WT ones, whereas the average root length of plants overexpressing *MORF9-GFP* did not differ significantly from that of WT plants (Figure 2b). Both yellowish rosette leaves and shortened roots during sugar-free germination were obvious in *morf9* mutant, which could be rescued by complementation of *Pmorf9:MORF9-myc* (Figure 2a,b). The measurement of leaf photosynthetic parameters indicated that the initial fluorescence parameter F0 of the 3^th^ leaf of *morf9* plants 12 days after germination was significantly higher than that of WT plants (Figure 2c), whilst their maximum photochemical efficiency Fv/Fm was the lowest among the four lines (Figure 2d). Complementation by *Pmorf9:MORF9-myc* restored the F0 and Fv/Fm values of *morf9* plants to those of WT plants. No significant difference in the values of Fv/Fm between the overexpression line *MORF9-GFP* and WT plants was observed. Therefore, the loss of *MORF9* had a negative effect on leaf photosynthetic efficiency and rendered the chloroplasts more sensitive to light.

**Figure 3:**
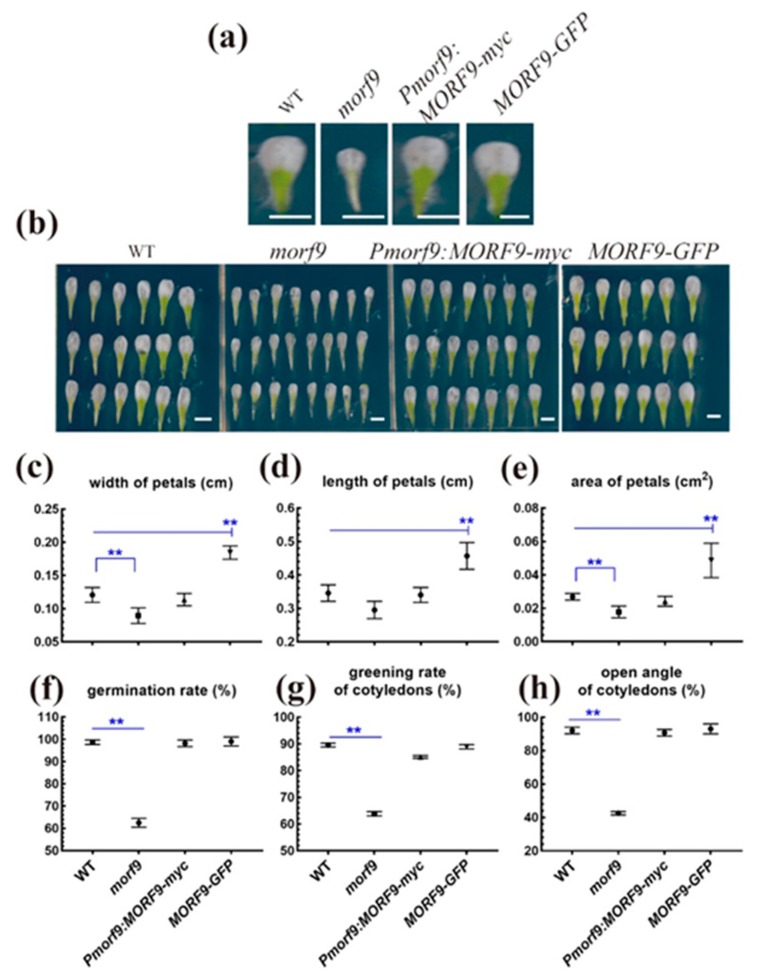
Flower phenotype and seed germination in the *morf9* mutant and *MORF9* transgenic plants. (**a**,**b**) Representative images of flowers in 56-day-old plants. Scale bars = 10 mm; (**c**–**e**) Flower dimensions of the indicated plant lines; *n* = 18–24; (**f**) Seed germination rate on the 5th day. Three independent experiments with *n* = 1500; (**g**) Cotyledon greening rates in the 5-day-old plants. Three independent experiments with 100 seedlings each; (**h**) Open cotyledons in 5-day-old plants. Three independent experiments with 100 seedlings each. Error bars indicate the standard errors of the replications. Asterisks indicate significant differences from the WT according to two-tail Student’s t test (* denotes *p* < 0.05, ** for *p* < 0.01 and *** for *p* < 0.001).

The loss of *MORF9* also had an impact on the size of the floral organs. In *morf9* plants, the floral organs were significantly smaller than in WT plants. The overexpression line had the largest flowers, whereas the complementation line could rescue the phenotype of *morf9* plants (Figure 3a–e). Thus, the loss of *MORF9* seemed to reduce the flower’s size as well.

Furthermore, the germination rate, greening rate of cotyledons, and open angle of cotyledons were measured in 5-day-old plants grown on sugar-free 1/2 MS medium. The results showed that the germination rate of the seeds and the cotyledon greening rate and open angle in the *morf9* line were significantly lower than in WT plants. As expected, no difference was observed between the *MORF9-GFP* line and the WT line. In *Pmorf9:MORF9-myc* plants, this phenotype of *morf9* plants was completely rescued (Figure 3f–h).

### 2.2. Expression and Distribution of MORF9 in Different Tissues and Organs

**Figure 4:**
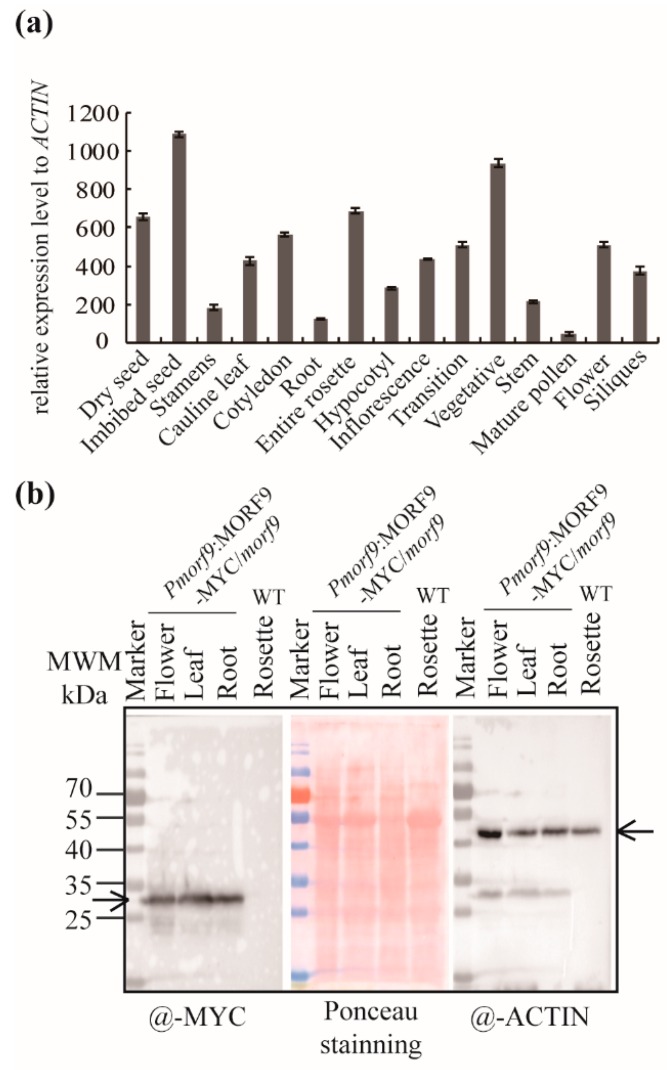
*MORF9* transcript and protein levels in various tissues and organs. (**a**) The transcript levels of *MORF9* in various tissues and organs retrieved from public datasets [33]; (**b**) Protein levels of MORF9 in various organs in *Pmorf9:MORF9-myc* plants, determined by using an antibody against MYC. The same membrane was subsequently probed with an anti-ACTIN antibody for loading control. Ponceau staining prior to immunodetection shows total proteins on the membrane. Arrows indicate the expected bands.

The microarray data taken from public datasets on The *Arabidopsis* Information Resource (TAIR) website [33] showed that *MORF9* was highly expressed in the imbibed seeds, rosettes, and shoot apex but lowly expressed in roots, stamens, pollens, and stems (Figure 4a) [33]. Using the complementation line expressing MYC-tagged MORF9, we monitored the protein levels in different tissues and organs with an antibody against MYC (Figure 4b). The immunodetection showed a high accumulation of MORF9 proteins in the roots and leaves and a slightly low accumulation in flowers, when normalized to the ACTIN control (Figure 4b). The immunodetection results seemed not consistent with the microarray data, implicating a likely post-transcriptional regulation or RNA turnover control mechanism for *MORF9*.

### 2.3. RNA Editing Efficiencies Associated with MORF9 Were Altered in Various Tissues and Organs

Since the loss of MORF9 caused retardation in the development of root, leaf, flower, and seed, in order to explore whether this phenotypical effect was related to MORF9-associated RNA editing efficiency, we analyzed multiple transcripts of the genes known to be edited by MORF9 [26] in various tissues and organs, in both *morf9* and WT lines, using the bulk-cDNA sequencing technique.

#### 2.3.1. Alteration of Plastid RNA Editing Efficiency in the Leaves of the *morf9* Mutant

**Figure 5:**
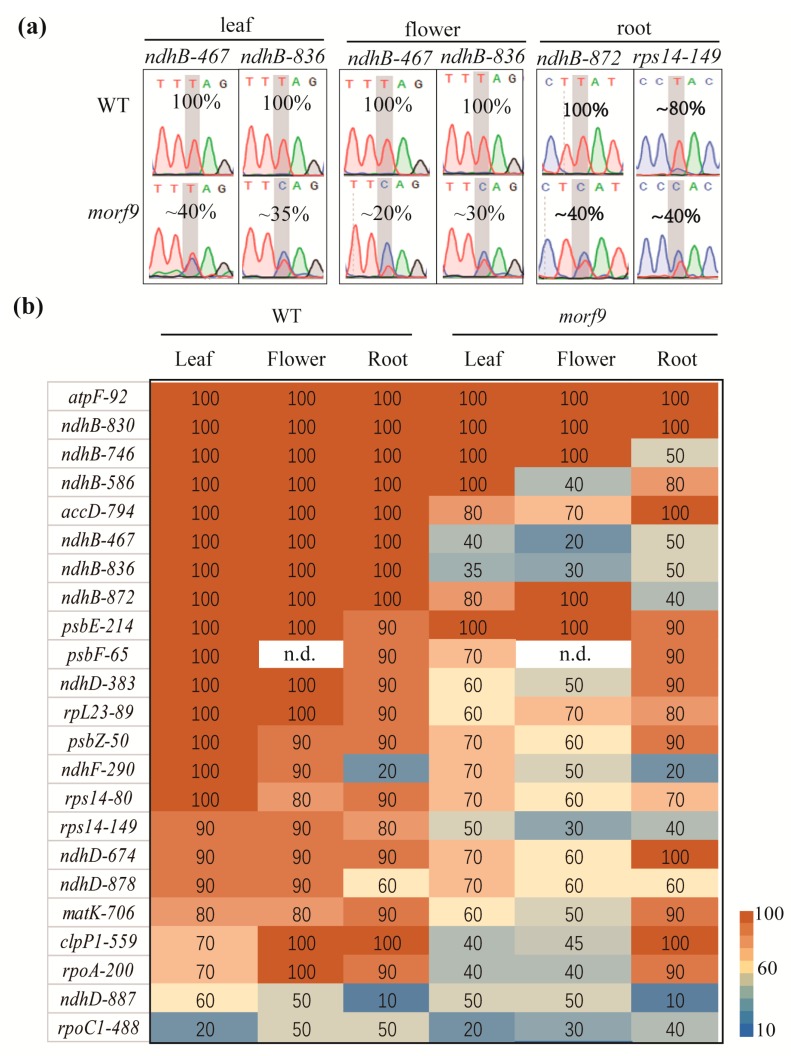
Percent editing of 24 plastid RNA sites in various organs of WT and *morf9* seedlings. (**a**) Nucleotide sequence profiles of the RNA editing sites in leaf, flower, and root most significantly affected by *morf9* mutation; (**b**) A heatmap of RNA editing efficiencies for multiple editing sites in various organs of WT and *morf9* plants. Leaf and root samples were collected in seedlings after 21 days of growth in sugar-free medium. For flower sampling, 56-day-old plants were used.

The WT and *morf9* plants were grown on sugar-free medium for 21 days. The editing efficiency of 24 plastid RNA editing sites in rosette leaves was determined using a similar method as that reported by Li et al. [26]. The results showed that the editing efficiency of the 6 sites *atpF-92, ndhB-830, ndhB-746, ndhB-586*, *psbE-214*, and *rpoC1*-*488* in the *morf9* mutant was comparable to that of the WT line, while the editing efficiency of the other 17 sites decreased by approximately 20% (*accD*-*794*)–65% (*ndhB*-*836*) compared to that of the WT control (Figure 5b, Supplemental Figure S2). The largest differences were detected for *ndhB-467* and *ndhB-836* (Figure 5a).

#### 2.3.2. Plastid RNA Editing Efficiency in Flowers of the *morf9* Mutant

In order to investigate whether the loss of MORF9 affects the editing efficiency of multiple RNA editing sites in flowers, we checked flowers transcripts for the 23 editing sites in both *morf9* mutant and WT plants. The editing of transcripts in the six plastid RNA sites *atpF-92*, *ndhB-830, ndhB*-*746*, *ndhB-872, psbE-214,* and *ndhD*-*887* was not altered in the *morf9* mutant. However, the editing efficiency of the other 16 transcript sites was reduced from ~30% (*accD*-*794* and *rpL23*-*89*) to 80% (*ndhB*-*467* and *ndhB-836*) in the mutant plant (Figure 5b, Supplemental Figure S2). The editing sites *ndhB-467, -586,* and *836* presented the most extensive changes (Figure 5a). Interestingly, even for WT plants, the RNA editing efficiencies of *rpoC1-488, rpoA-200*, and *clpP1-559* in the leaves were quite different from those in flowers (Figure 5b). Thus, although MORF9 participated in plastid RNA editing in both flower and leaf tissues, there were still some subtle differences in its activity in leaves and flowers. For example, the loss of MORF9 had no effect on *rpoC1-488* and *ndhB-586* editing in the leaves but reduced their editing efficiencies in the flowers. The editing efficiency at the site *ndhB-872* remained unchanged in the flowers but decreased in the leaves of the *morf9* mutant (Figure 5b).

#### 2.3.3. Detection of Plastid RNA Editing Efficiency in the Roots of the *morf9* Mutant

Next, we further compared the editing efficiency of plastid RNA in the roots between the *morf9* mutant and WT plants. Among the 23 editing sites, 13 were not affected in their editing efficiencies by the *morf9* mutation. These included *accD-794, atpF-92, clpP1-559, matK-706, ndhB-830, ndhD-383, ndhD-878, ndhD-887, ndhF-290, psbE-214, psbF-65, psbZ-50*, and *rpoA-200*. The remaining 10 sites were edited with altered efficiencies in *morf9* and WT plants (Figure 5b, Supplemental Figure S2). The most reduced editing efficiency was found at *ndhB*-*872* site, which had an editing rate of 40% in the *morf9* mutant, while was 100% edited in WT plants (Figure 5a). The root seemed to have less plastid RNA editing events compared to leaf or flower in both WT plants and *morf9* mutant (Figure 5b). Notably, the editing rate at sites *ndhD*-*887* and *ndhF*-*290* were only 10% and 20%, respectively, in the roots of both mutant and WT plants, whilst the values for other organs were 50% or above. These observations highlight differences in MORF9-mediated plastid RNA editing events between roots and rosette leaves or flowers.

**Figure 6:**
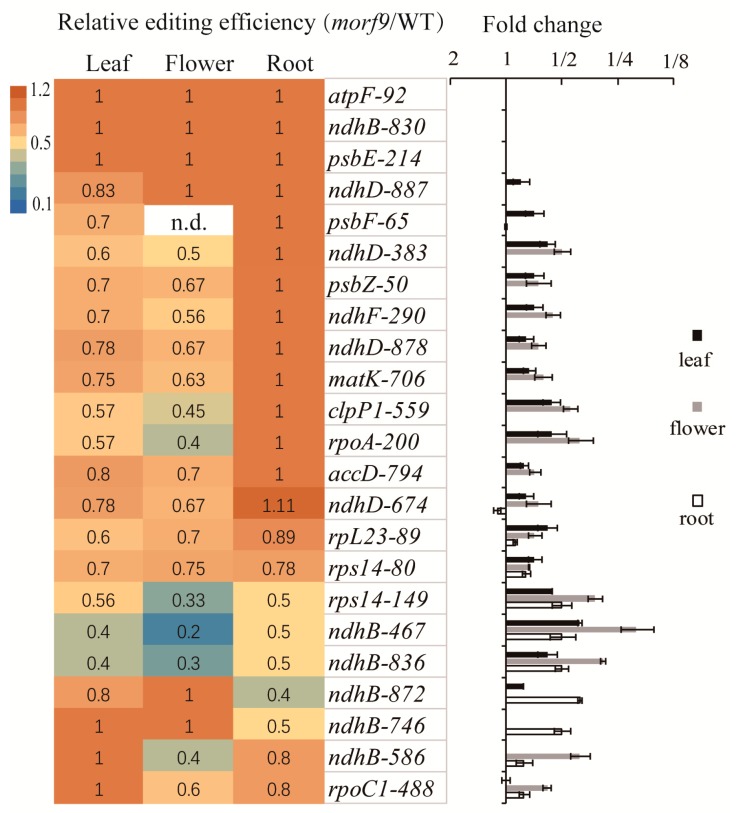
Summary of relative plastid RNA editing efficiency in the indicated organs in *morf9* plants as compared to WT plants. The left panel shows the average ratio of *morf9* mutant’s to WT line’s editing efficiency from three replicates. The right panel plots the fold change relative to the WT, showing standard errors of the replicates. Full dataset are provided in Supplemental Table S2.

Taken together, the loss of MORF9 protein decreased the editing efficiency of various plastid transcripts at multiple sites in flowers, rosette leaves, and roots (Figure 6). In the three tissues, four sites *atpF-92, ndhB-830, psbE-214* and *ndhD-887* were not affected by the loss of MORF9, whereas the editing efficiencies at sites *rps14*-*80*, *rps14*-*149*, *ndhB*-*467*, and *ndhB*-*836* in these tissues were reduced in an apparently similar way by the mutant (Figure 6). Furthermore, the loss of MORF9 specifically altered the editing of the *psbF-65* site in leaves, of the *ndhB-746* in roots, and of the 9 sites *ndhD-383, psbZ-50, ndhF-290, ndhD*-*878*, *matK-706, clpP1-559, rpoA-200, accD-794*, and *ndhD-674* in both leaf and flower tissues (Figure 6). In addition, a reduced editing caused by *morf9* mutation at sites *ndhB-586* and *rpoC1-488* was observed in flowers and roots only, whilst the largest decreased in the editing efficiency found in roots was at site *ndhB*-*872*, whose editing efficiency was also reduced, though to a much less extent, in leaves - but not in flowers (Figure 6).

### 2.4. Alteration of RNA Editing Efficiency during Leaf Aging

**Figure 7:**
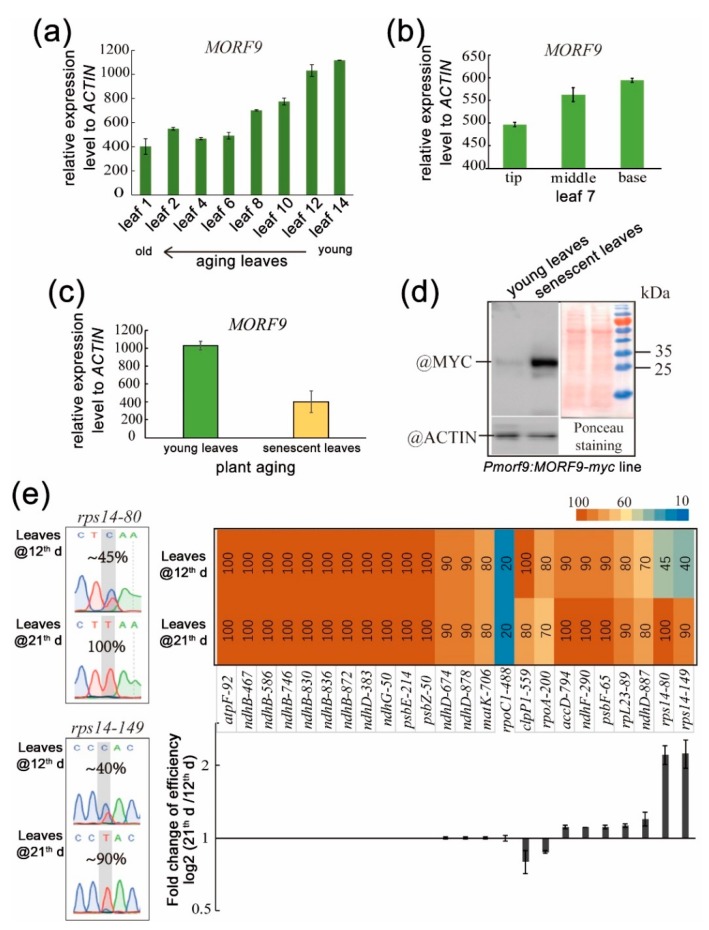
Alteration of *MORF9*-associated RNA editing efficiency during leaf senescence in WT *Arabidopsis*. (**a**) *MORF9* mRNA levels of subsequent rosette leaves of mature plants; (**b**) *MORF9* mRNA levels of leaf 7; (**c**). *MORF9* mRNA levels of rosette leaves of young and aging plants; (**d**). Western blot of MORF9-MYC fusion protein in rosettes of plants expressing the tagged MORF9 driven by the native promoter. The Ponceau staining of the same membrane and Western blotting of ACTIN by anti-ACTIN antibody were used to control gel loading; (**e**) Plastid RNA editing efficiency in leaves of 12-day-old and 21-day-old plants. Full dataset are provided in Supplemental Table S2.

Data taken from the published NASCArrays information (http://www.bar.utoronto.ca/NASCArrays/) showed that during plant development, *MORF9* expression level tended to decline with leaf senescence and plant aging (Figure 7a–c). From these data, it could be estimated that senescent or mature leaves had approximately two-fold lower mRNA levels than young leaves. However, the protein level of *MORF9* ectopically expressed from its native promoter accumulated more abundantly in senescent leaves (Figure 7d). It seemed that *MORF9* mRNA level was not correlated to the protein level in leaf tissue during senescence or aging. Nevertheless, in WT *Arabidopsis*, we still observed moderate differences in RNA editing in the known MORF9-associated sites during leaf aging (Figure 7e). Among the transcripts of 24 known genes, 2 sites (*clpP1*-*559* and *rpoA*-*200*) showed a small decrease in the editing rate in leaves of 21-day-old plants compared to 12-day-old plants, whilst 5 sites (*accD-794, ndhF-290, psbF-65, rpL23-89*, and *ndhD-887*) had slightly more editing efficiency in older leaves (Figure 7e, Supplemental Figure S2). A notable increase in editing associated with aging was observed at the sites *rps14*-*80* and *rps14*-*149*, thus the transcript of this gene was affected during leaf senescence (Figure 7e).

## 3. Discussion

RNA editing often results in modified amino acid sequences of mature proteins, by creating an initiation codon from ACG or correcting codons of evolutionary conserved amino acid residues [34]. Mutants defective in RNA editing show loss of function of the target gene products. These mutations are sometimes linked to severe phenotypic disorders [16,35,36]. MORF9 is a pivotal factor of the labile plastid RNA editosome complex and participates in a wide range of editing events at multiple sites of plant plastid RNAs. In this study, we provide further evidence that MORF9-mediated RNA editing is tissue-dependent and the resultant organelle proteomes are pertinent to the specific tissue function. The known editing sites associated with MORF9 were all located in the exon regions of the genes [29], whose alterations caused by the loss of MORF9 resulted in conversions of amino acid residues (Table 1).

The outcomes of RNA editing are well known to correlate with plant growth and development and to be influenced by environmental changes [37,38,39,40,41]. MORF9-assocated RNA editing events of plastid genes showed similarity in leaves and flowers but displayed a remarkable difference between roots and leaves or flowers (Figure 5). This interesting observation can be explained by the closeness in organ origin between leaves and flowers. On the other hand, RNA editing of the *psbF* gene was affected by the loss of MORF9 only in the leaves and not in the flowers and roots (Figure 5 and Figure 6). The *psbF* gene encodes the β-subunit of cytochrome b559, an important component of photosystem II in the photosynthetic tissue [31,42], and a reduced transcript editing rate of 30% caused by *morf9* mutation in this case (Figure 5) may have some unknown effects on the gene product’s performance, although it is still difficult to identify the effects of the mutant. Nevertheless, it is interesting to see that the *Arabidopsis morf9* mutant showed a very similar phenotype to the tobacco *psbF* mutant plant, in which the alteration of multiple RNA editing sites of *psbF* leads to a phenotype of pale green leaves, reduces photosynthetic efficiency, and delays growth and development [43,44].

Organ-specific alteration in MORF9-mediated RNA editing is possibly linked to the conserved or specific functions of the target genes. Two genes whose RNA editing was not affected in any tissues by the loss of MORF9 are *atpF* and *psbE*, (Figure 6). The gene *atpF* encodes an essential chloroplast ATP synthase subunit, while *psbE* is a conserved gene for photosystem II in most photosynthetic organisms. RNA editing of both genes has also been shown to require MORF2 (RIP2) [29]. Therefore, other editing factors may compensate for the absence of MORF9 for proper transcript editing of essential proteins. Another gene, *ndhB*, which encodes NADH dehydrogenase subunit 2 and plays a role in oxidative phosphorylation, has multiple editing sites, among which only site *C830* was not affected in the *morf9* mutant, whilst *C746* site editing was specifically reduced by half in the roots (Figure 6 and Table 1). Furthermore, reduction in editing by the loss of MORF9 was observed in both flowers and roots at *ndhB*-*586*, whilst it was found only in leaves and roots at *ndhB-872,* with more than one-fourth decrease of editing efficiency in the roots (Table 1 and Figure 6). Thus, in different organs, the editing sites of the *ndhB* transcript are diversely affected (Table 1). It has been reported that interactions of MORF9 with other editing factors may influence which sites are edited and the editing efficiency at *ndhB*, leading to changes in NDH activity [21]. Overall, the observed organ-specific profiling in MORF9-associated editing is likely a reflection of the activity of the versatile RNA editing complex in different organs [16], and one could expect that a mixture of the resulting heterogeneous, edited proteins might have an impact on organs’ functions.

Moreover, we observed that low transcript levels of *MORF9* did not necessarily result in low proteins levels during organ aging or in various tissues, i.e., low transcript levels but high accumulation of proteins in aging leaves (large difference from young leaves) or in roots (small difference from other tissues) were found (Figure 4 and Figure 7c–d), meaning that MORF9 protein turnover or stability is differently controlled during aging or in different tissues. The RNA editing efficiency seemed to be correlated with MORF9 protein amount. For example, the RNA editing efficiency of *accD*-*794*, *ndhF-290*, *psbF-65*, *rpL23-89*, *ndhD-887*, *rps14-80*, and *rps14-149* increased to various extents with leaf aging when MORF9 protein level increased. Among these affected genes, *rps14* has been reported to be associated with leaf senescence [8,45]. However, the involvement and significance of tissue-dependent RNA editing by MORF9 in organ senescence await further clarification.

In summary, MORF9 functions in plastid RNA editing with various efficiency during plant development and in in different tissues, which is at least partly due to (1) regulated MORF9 availability at both transcriptional and translational levels; (2) tissue-specific MORF9-interacting partners such as PPRs or others; (3) alternative RNA editors differently organized.

## 4. Materials and Methods

### 4.1. Plant Materials and Growth Conditions

WT plants were *Arabidopsis thaliana* Columbia ecotype (Col-0). A *T-DNA* insertion *morf9* mutant with Col-0 background was purchased from SALK Institute and screened for homozygous genomic insertion using specific primers suggested by the provider (http://signal.salk.edu/). *MORF9* gene expression was confirmed via qRT-PCR. Primers used are listed in supplemental Table S1. The homozygous insertion line was used for phenotyping and for the generation of complementation transgenes.

*A. thaliana* plants were maintained in a climate room with a temperature of 23 °C and a humidity of 60%, under a light intensity of 70 micromol⋅s^−1^·m^−2^ and light/dark cycle of 16/8 h.

Plants grown in 1/2 MS medium for 12 days after germination were used for measurement of root length. This was done by imaging in a photo scanner (Epson Perfection V600, Epson (China) Co., Ltd., Beijing, China) and quantification using the Image J software (version 1.51j8). At least 10 to 20 plants were recorded. Plants grown in soil for 56 days after germination were used to measure flower size, including the length and area of 20 flowers. Seed germination was scored on 500 seeds sown in square plates (10 × 10 cm), with three replicates. Cotyledons were scored for greening rate and open angle on 100–300 seedlings.

#### 4.2. qRT-PCR and Plastid RNA Editing Efficiency Analysis

Samples were taken from seedlings grown in 1/2 MS medium for 12 days (roots and leaves) and 21 days (leaves) after germination, and from seedlings grown in pots for 56 days (flowers) after germination. Samples were immediately frozen in liquid nitrogen and stored in a refrigerator at −80 °C. Total RNA was extracted from grounded tissues in liquid nitrogen using the TRIzol^®^ Plus RNA Purification Kit from Invitrogen™ (Thermo Scientific™, Shanghai, China) and digested by DNaseI. First-strand cDNA was synthesized with the RevertAid First-Strand cDNA Synthesis Kit (Thermo Scientific™, Shanghai, China). All qPCR reactions were performed in triplicate for each biological replicate on a Roche Light Cycler 96, using a PCR program set as follows: an initial step of 30 s at 94 °C, followed by 40 cycles of 15 s at 94 °C, and 30 s at 60 °C. The relative expression levels of genes were calculated by the 2 ^(−ΔΔC(T))^ method [46], using *ACTIN2* as an endogenous reference gene (primers in Table S1).

Using the above cDNAs as templates, the PCR products of the corresponding plastid genes were amplified with gene-specific primers (Table S1) and sent to a company (Shanghai Boshan Biotechnology Co., Ltd., Shanghai, China) for sequencing. The PrimeSTAR Max DNA Polymerase high-fidelity system (Takara Biomedical Technology Co., Ltd., Beijing, China) was used for PCR amplification. On the basis of the sequencing results, the editing efficiency was analyzed according to the sequencing profiles, as detailed in Chateigner-Boutin [47] and Germain [48]. The editing efficiency of each site was obtained by measuring the relative peak height of the nucleotide in the corresponding chromatograms and calculating the percentage of the height of “T” with respect to the sum of the height of “T” and “C”. The experiments were conducted three times. The chromatograms are provided in Supplemental File 1.

### 4.3. Construction of MORF9-Transgenic Plants

The complementation *Arabidopsis* line *ProMORF9:MORF9-4xMYC* was kindly provided by Dr. Yang Zhongnan’s Laboratory of Shanghai Normal University [30]. A binary vector for MORF9-2xFlag-GFP overexpression was constructed, based on the modified pCMBI33-2xflag-GFP vector [49]. The *MORF9* coding sequence was PCR-amplified with primers listed in Table S1 and cloned into the vector downstream of the *ACTIN* promoter in fusion with C-terminal 2xflag-GFP. All constructs were verified by sequencing.

*Agrobacterium tumefaciens GV3101* strain harboring the above overexpression plasmids was used for transformation of Col-0 plants by the floral dip method. T0 transgenic plants were selected using Basta herbicides (1/1000 concentration). *In planta* expression of the fusion protein was monitored under a stereoscopic fluorescence microscope (Leica M205 FA, Leica Microsystems, Shanghai, China), and fluorescent seedlings were collected to detect the size of the fusion protein by Western blot (Figure S1). T1 Plants with segregation ratio 3:1 were screened to obtain single-insertion homozygous transgenic plants.

### 4.4. Protein Detection of MORF9 in Transgenic Plants

Proteins were extracted from rosette leaves, flowers, and roots according to the phenol method [50]. Protein concentration in the extracts was determined by the Bradford method [51]. Proteins were separated on 12.5% polyacrylamide gels [52] and transferred to PVDF membranes by semi-dry electroblotting. Immunodetection was done according to protocols described in reference [53], using commercial antibodies against GFP (TransGen Biotech, Beijing, China) and MYC (CoWin Bioscience, Beijing, China).

### 4.5. Detection of the Efficiency of Photosystem II (Fv/Fm) and Initiation Fluorescence Yield (F0)

The maximum photochemical efficiency of photosystem II (Fv/Fm) and the minimal initiation fluorescence yield (F0) values were measured using Imaging-PAM-Maxi (Heinz Walz GmbH, Effeltrich, Germany) as described by Guan [54]. Briefly, plants were adapted to darkness for 30 min prior to being exposed to a light intensity of 54 μmol·m^−2^s^−1^ photosynthetically active radiation (PAR), and the kinetic curves were recorded according the instruction of the instrument. The values of Fo and (Fv/Fm) were calculated through one saturation pulse. Leaf 3 from individual plants, 21 day after germination, were measured, with 3 points on each leaf from at least 5 individual plants. Fo was measured at a low light intensity, and the maximal fluorescence yield (Fm) was measured under saturation pulse. The chlorophyll fluorescence images of whole plants were taken by Imaging-PAM-Maxi.

### 4.6. Statistical Analysis

Where appropriate, either two-way ANOVA or pair-wide multiple t-tests on the means of at least three biological replicates was performed in Microsoft Excel (version 2013). Data are shown as mean ± SE.

## Figures and Tables

**Table 1:** Organ-dependent editing alterations in plastid genes encoding RNAs and proteins by loss of MORF9 in *A. thaliana.*

Gene	Edited Nucleotide	Amino Acid Change	Reduced Editing Found Organs	Annotation
*accD*	*C794*	S265–L265	Leaf and flower	acetyl-CoA carboxylase beta subunit
*clpP1*	*C559*	H187–Y187	Leaf and flower	ATP-dependent ClpP1 protease
*matK*	*C706*	H236–Y236	Leaf and flower	a splicing factor, Maturase K
*ndhB*	*C586*	H196–Y196	Flower and root	NADH dehydrogenase subunit
*ndhB*	*C746*	S249–F249	Root	NADH dehydrogenase subunit
*ndhB*	*C872*	S291–L291	Root and leaf	NADH dehydrogenase subunit
*ndhD*	*C887*	P296–L296	Leaf	NADH dehydrogenase subunit
*ndhD*	*C383*	S128–L128	Leaf and flower	NADH dehydrogenase subunit
*ndhD*	*C674*	S225–L225	Leaf and flower	NADH dehydrogenase subunit
*ndhD*	*C878*	S293–L293	Leaf and flower	NADH dehydrogenase subunit
*ndhF*	*C290*	S97–L97	Leaf and flower	NADH dehydrogenase subunit
*psbF*	*C65*	S26–F26	Leaf	photosystem II
*psbZ*	*C50*	S17–L17	Leaf and flower	photosystem II
*rpoA*	*C200*	S67–F67	Leaf and flower	RNA polymerase
*rpoC*	*C488*	S163–L163	Flower and root	RNA polymerase

## Supplementary Materials

Supplementary materials can be found at https://www.mdpi.com/1422-0067/20/18/4635/s1. Supplemental Figure S1: Molecular identification of *morf9* mutant and overexpression lines; Supplemental Figure S2: Nucleotide sequence profiles in RNA editing transcripts; Supplemental Table S1: List of the primers used in the experiment; Supplemental Table S2: Dataset of efficiency of plastid RNA editing sites
