# Supplementary material for: MORF9 Functions in Plastid RNA Editing with Tissue Specificity
Source: Int J Mol Sci. 2019 Sep 19;20(18):4635. doi: 10.3390/ijms20184635 (PMC6769653; doi:10.3390/ijms20184635)
Supplement: Supplementary file 1 [file ijms-20-04635-s001.zip › Supplemental Table S1-List of primers used in the experiment.pdf]

Supplemental Table S1 List of the primers used in the experiment

	Primer	sequence	usage	Amplicom size (bp)
1	*accD-FP*	ATGGAAAAATCGTGGTTCAATTTTATGTT	RNA editing	400
	accD-RP	GTTTGTCTAGTCTAATTTGAACTTCCCC	RNA editing	
2	*atpF-FP*	TTAACCGATTCTTTCGTTTACTTG	RNA editing	555
	atpF-RP	CAAACATCCCAATATTTGCATTAA	RNA editing	
3	*clpP -FP*	TATTGGCGTTCCAAAAGTACCTT	RNA editing	591
	clpP -RP	GAACCGCTACAAGATCAACAATTC	RNA editing	
4	*matK-FP*	ATAAATTTCAAGGATATTTAGAGTTCG	RNA editing	382
	matK-RP	TTGACCAAATCATTAAGATAAAGAATA	RNA editing	
5	*ndhB-FP*	GCATGTACAGAATGAAAATTTCATTCTC	RNA editing	1168
	ndhB-RP	AATCGCAATAATCGGGTTCATT	RNA editing	
6	*ndhD-FP*	AACAACTCGAAGTATGGGTC	RNA editing	1083
	ndhD-RP	CTAATGAGAGCACAAAATCAGGAT	RNA editing	
7	*ndhF-FP*	GGATCATACCTTTCATTCCACTTC	RNA editing	471
	ndhF-RP	GCAGCATGTATAAGAGCCGAAATG	RNA editing	
8	*ndhG-FP*	TTTGCCTGGACCAATACATG	RNA editing	531
	ndhG-RP	AGCCACAGAAATTGCACCTAT	RNA editing	
9	*psbE-FP*	ATGTCTGGAAGCACAGGAGA	RNA editing	252
	psbE-RP	CTAAAACGATCTACTAAATTCATCGAG	RNA editing	
10	*psbF-FP*	GATAGGACCTATCCAATTTTTACAG	RNA editing	120
	psbF-RP	CGTTGGATGAACTGCATTGC	RNA editing	
11	*psbZ-FP*	TGCTTTCCAATTGGCAGTTTT	RNA editing	178
	psbZ-RP	TCAAGAGATAAGAGAATTAAGGATACC	RNA editing	
12	*rpl23-FP*	ATGGATGGAATCAAATATGC	RNA editing	264
	rpl23-RP	TTAAGTTCTTTTCTTTCTAAGAGG	RNA editing	
13	*rpoA F2-FP*	CTCGGACACTACAGTGGAAGTGTG	RNA editing	253
	rpoA F2	TTCTACGTGAAAATGTTCAATTTTGATAAG	RNA editing	
14	*rpoB 65F-FP*	GGTTTTATCGGTTTATTGATCAGGG	RNA editing	2019
	rpoB 65F-RP	CGGCGACCAATCCTTCCTAATTCAC	RNA editing	
15	*rpoB-FP*	TTCAGGTATCGACTTCAAAAGAAAC	RNA editing	2019
	rpoB-RP	GGATACTCGGGTTCAAATACCC	RNA editing	
16	*rpoC1-FP*	CCTACTTTCTTACGATTACGAGGTT	RNA editing	1737
	rpoC1-RP	ATGGGTCTCAACTCGGGAGG	RNA editing	
17	*M9-1-LP*	AGATGCTTCTATTCTTCCCCC	Mutant screen	
	M9-1-RP	ATTGTGTCCTGATTTCGTTGG	Mutant screen	
18	*LBb1.3/LP*	ATTTTGCCGATTTCGGAAC	Mutant screen	
19	*M9-FP*	ATGGCTTCCTTCACAACAACT	qRT-PCR	
	M9-RP	TCTTCCATGCTGCCAAGAACA	qRT-PCR	
20	*MORF9-iGFP-F*	TGACCTCGAGACTAGTATGGCTTCCTTCAC AACAAC	subclone	
	MORF9-iGFP-R	AGGTGGAGGTCCCCCGGGAGAGGAATCAG AGGCTGCTG	subclone	
