# Supplementary figures and images for: MORF9 Functions in Plastid RNA Editing with Tissue Specificity
Source: Int J Mol Sci. 2019 Sep 19;20(18):4635. doi: 10.3390/ijms20184635 (PMC6769653; doi:10.3390/ijms20184635)

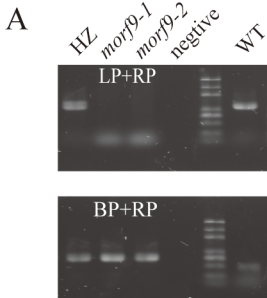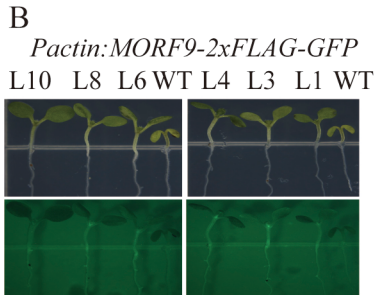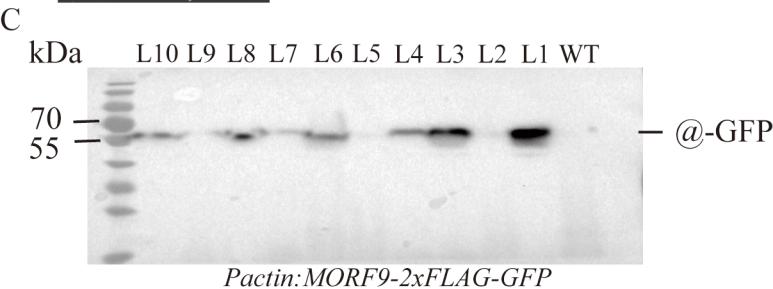

Supplement: Supplementary file 1 [file ijms-20-04635-s001.zip › Supplemental Figure S1-molecular identification of morf9 mutant and overexpresion lines.pdf]

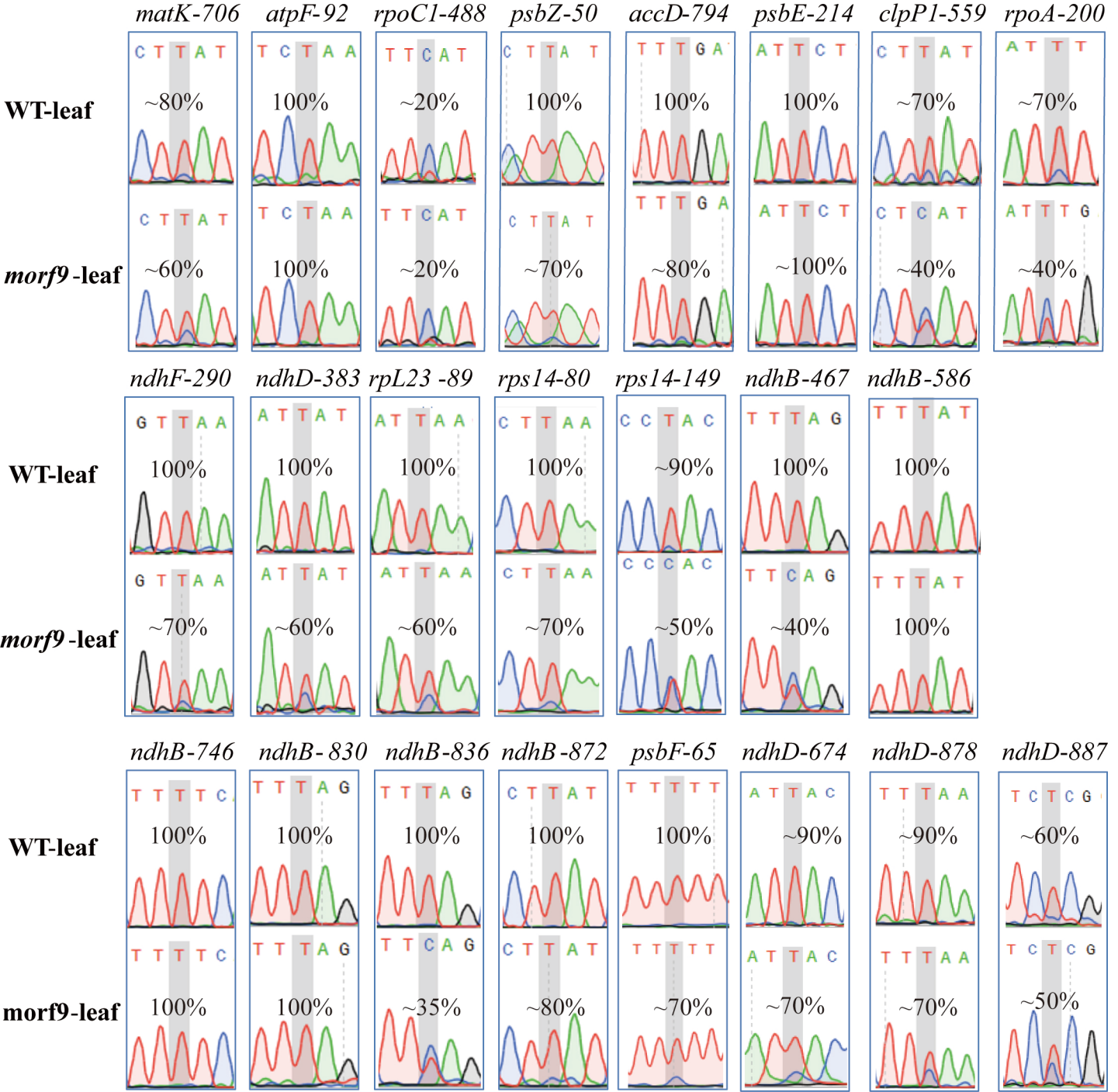

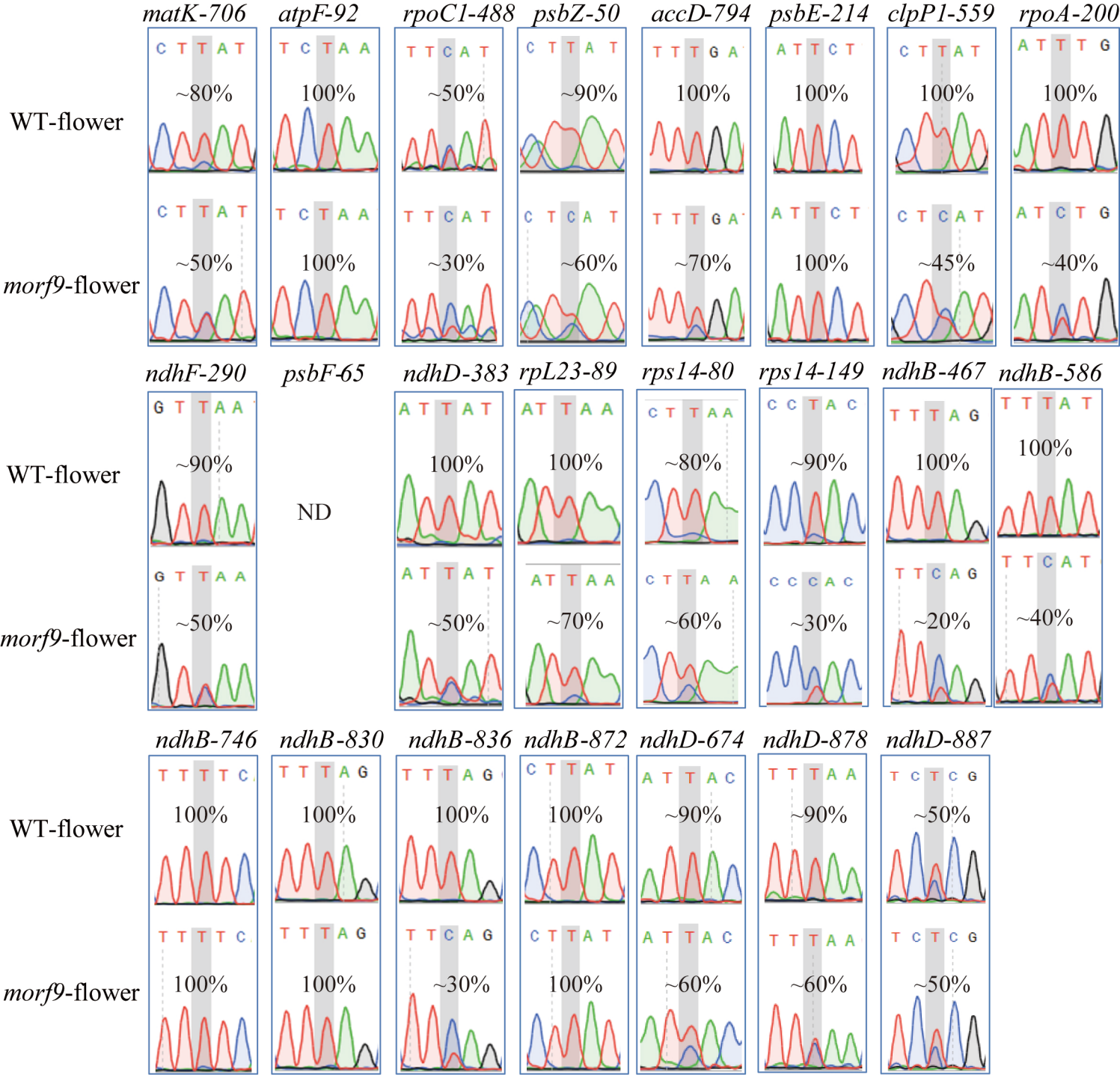

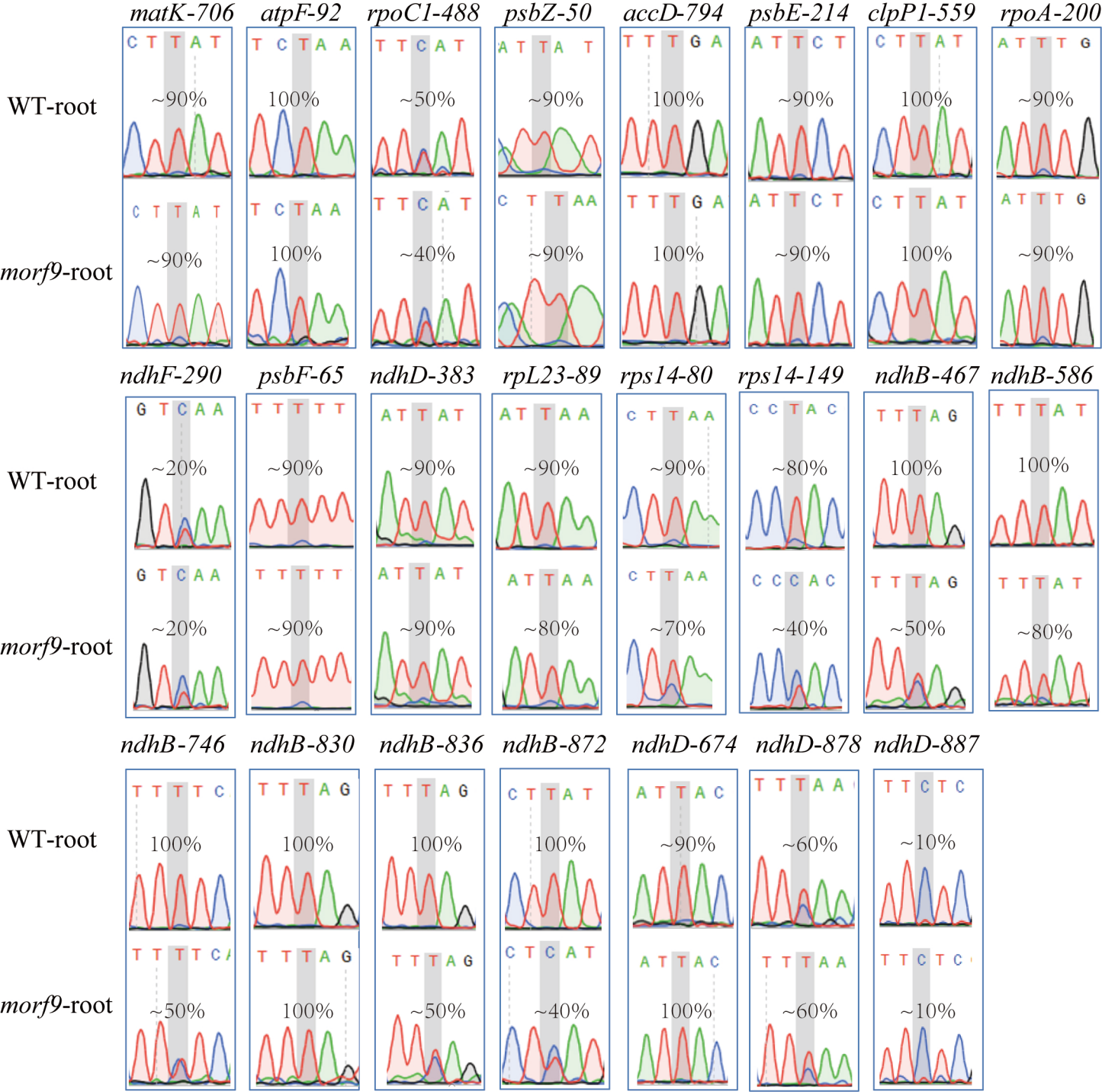

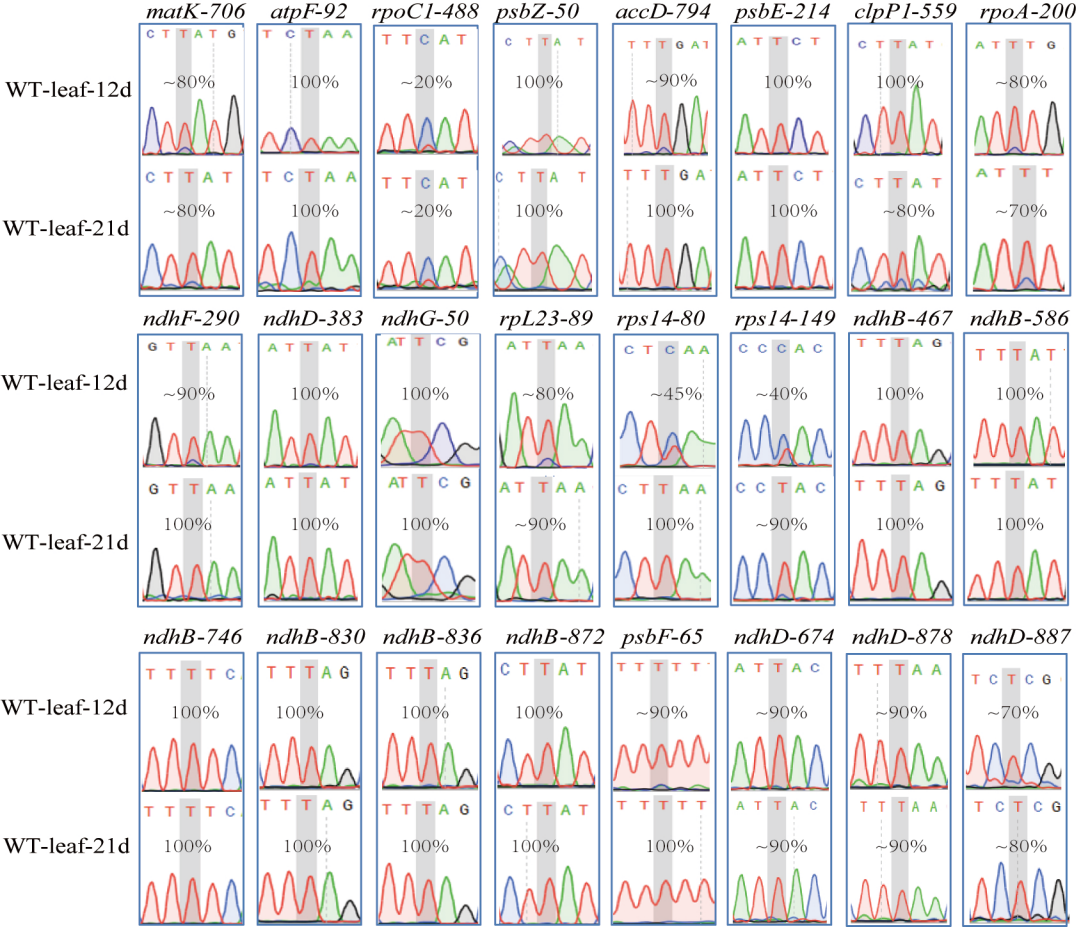

Supplement: Supplementary file 1 [file ijms-20-04635-s001.zip › Supplemental Figure S2 Nucleotide sequence profiles in RNA editing transcripts.pdf]
